# Localisation cutanée du lymphome de Manteau

**DOI:** 10.11604/pamj.2013.16.109.3469

**Published:** 2013-11-21

**Authors:** Naoufal Hjira, Mohammed Boui

**Affiliations:** 1 Service de Dermatologie, Hôpital Militaire d'Instruction Mohammed V, Rabat, Maroc

**Keywords:** Lymphome de Manteau, lymphome B, ganglion, Mantle cell lymphoma, B cell lymphoma, lymph node

## Image en medicine

Individualisé en 1992, le lymphome de manteau est un lymphome B rare, développé au dépend des cellules du manteau folliculaire des centres germinatifs des ganglions, touchant le sujet âgé, souvent disséminé avec splénomégalie, envahissement médullaire et tropisme particulier pour les muqueuses digestives. Les cellules lymphomateuses expriment des marqueurs pan B, elles sont CD5 positives et CD23 négatives. Une translocation t (11,14) (q13, q32) est mise en évidence dans 50 à 80% des cas. Le recours à la chimiothérapie conventionnelle, intensive et à l'immunothérapie (anticorps anti CD20) ne semble pas améliorer le pronostic, l'évolution se fait par détérioration progressive avec réponse habituellement temporaire au traitement. Nous rapportons un cas original d'un patient âgé de 46 ans, présentait dans un contexte de fièvre, d'asthénie, une polyadénopathie périphérique (sus claviculaire, jugulaire droites et axillaires) associés à des multiples nodules sous cutanés, siégeant sur les membres supérieurs, inférieurs et sur l'abdomen, de consistance ferme, mesurant 1 à 3 cm de diamètre. L'examen histologique d'un ganglion et d'un nodule cutané, a conclu à un lymphome non hodgkinien à petites cellules dont l'immunophénotypage était positif pour CD5 et CD20 et négatif pour CD23. Le diagnostic de lymphome de manteau était retenu, le bilan d'extension réalisé a objectivé une infiltration lymphomateuse nodulaire à la biopsie ostéo-médullaire, une duodénite nodulaire à l'endoscopie digestive et une polyadénopathie profonde sur le scanner thoraco-abdominal. Le patient a été mis sous polychimiothérapie conventionnelle + anticorps anti CD20. L’évolution était marquée par l’échappement thérapeutique, nécessitant le recours à une chimiothérapie intensive.

**Figure 1:**
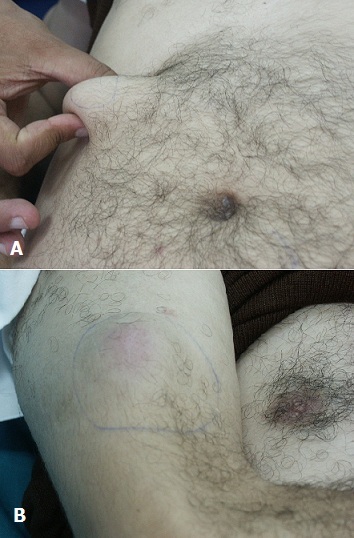
A) Nodule sous cutané abdominal; B) Nodule sous cutané du bras droit

